# Supplementary material for: Heparin-based hydrogel scaffolding alters the transcriptomic profile and increases the chemoresistance of MDA-MB-231 triple-negative breast cancer cells
Source: Biomater Sci. 2020 Feb 13;8(10):2786–96. doi: 10.1039/c9bm01481k (PMC7497406; doi:10.1039/c9bm01481k)
Supplement: Supplementary file 2 [file BM-008-C9BM01481K-s002.zip › Supplementary File 4/HP-B hydrogel + EGF (s)vControl/Pathways/my_analysis.Gsea.1545201170987/HALLMARK_INTERFERON_ALPHA_RESPONSE.html]

Details for gene set HALLMARK\_INTERFERON\_ALPHA\_RESPONSE[GSEA]

|  || Dataset | expr.class.cls#HYDEGF\_versus\_CONTROL.class.cls#HYDEGF\_versus\_CONTROL\_repos |
| Phenotype | class.cls#HYDEGF\_versus\_CONTROL\_repos |
| Upregulated in class | CONTROL |
| GeneSet | HALLMARK\_INTERFERON\_ALPHA\_RESPONSE |
| Enrichment Score (ES) | -0.53211266 |
| Normalized Enrichment Score (NES) | -2.259119 |
| Nominal p-value | 0.0 |
| FDR q-value | 0.0 |
| FWER p-Value | 0.0 |
Table: GSEA Results Summary

  

Fig 1: Enrichment plot: HALLMARK\_INTERFERON\_ALPHA\_RESPONSE      
 Profile of the Running ES Score & Positions of GeneSet Members on the Rank Ordered List

  

| PROBE | DESCRIPTION (from dataset) | GENE SYMBOL | GENE\_TITLE | RANK IN GENE LIST | RANK METRIC SCORE | RUNNING ES | CORE ENRICHMENT || 1 | LPAR6 | na |  |  | 664 | 1.935 | -0.0130 | No |
| 2 | TRIM14 | na |  |  | 1248 | 1.540 | -0.0263 | No |
| 3 | PARP12 | na |  |  | 1533 | 1.416 | -0.0252 | No |
| 4 | MOV10 | na |  |  | 2556 | 1.113 | -0.0662 | No |
| 5 | HERC6 | na |  |  | 2887 | 1.030 | -0.0719 | No |
| 6 | IRF7 | na |  |  | 2931 | 1.020 | -0.0627 | No |
| 7 | OASL | na |  |  | 3235 | 0.954 | -0.0679 | No |
| 8 | DHX58 | na |  |  | 3730 | 0.859 | -0.0841 | No |
| 9 | SLC25A28 | na |  |  | 4291 | 0.768 | -0.1048 | No |
| 10 | ISG15 | na |  |  | 4664 | 0.711 | -0.1163 | No |
| 11 | TRIM26 | na |  |  | 4988 | 0.653 | -0.1258 | No |
| 12 | MX1 | na |  |  | 5059 | 0.644 | -0.1223 | No |
| 13 | CMPK2 | na |  |  | 5304 | 0.604 | -0.1283 | No |
| 14 | TRIM25 | na |  |  | 5383 | 0.593 | -0.1257 | No |
| 15 | USP18 | na |  |  | 5430 | 0.586 | -0.1215 | No |
| 16 | OAS1 | na |  |  | 5434 | 0.585 | -0.1151 | No |
| 17 | IFI30 | na |  |  | 5554 | 0.570 | -0.1150 | No |
| 18 | RSAD2 | na |  |  | 5911 | 0.515 | -0.1278 | No |
| 19 | STAT2 | na |  |  | 6213 | 0.474 | -0.1382 | No |
| 20 | RNF31 | na |  |  | 6324 | 0.460 | -0.1388 | No |
| 21 | IFI44L | na |  |  | 6789 | 0.397 | -0.1586 | No |
| 22 | OGFR | na |  |  | 7200 | 0.343 | -0.1763 | No |
| 23 | IL7 | na |  |  | 7239 | 0.338 | -0.1744 | No |
| 24 | DDX60 | na |  |  | 7899 | 0.256 | -0.2061 | No |
| 25 | ADAR | na |  |  | 8616 | 0.170 | -0.2416 | No |
| 26 | UBA7 | na |  |  | 9109 | 0.113 | -0.2661 | No |
| 27 | TMEM140 | na |  |  | 9183 | 0.104 | -0.2687 | No |
| 28 | IFI35 | na |  |  | 9363 | 0.080 | -0.2772 | No |
| 29 | CNP | na |  |  | 9702 | 0.050 | -0.2943 | No |
| 30 | SAMD9L | na |  |  | 9711 | 0.048 | -0.2942 | No |
| 31 | SAMD9 | na |  |  | 9831 | 0.031 | -0.3001 | No |
| 32 | IFIT2 | na |  |  | 9862 | 0.027 | -0.3014 | No |
| 33 | IFI44 | na |  |  | 9863 | 0.027 | -0.3011 | No |
| 34 | IFIH1 | na |  |  | 10011 | 0.019 | -0.3085 | No |
| 35 | CASP8 | na |  |  | 11048 | -0.114 | -0.3615 | No |
| 36 | IRF1 | na |  |  | 11060 | -0.115 | -0.3608 | No |
| 37 | PNPT1 | na |  |  | 11210 | -0.124 | -0.3672 | No |
| 38 | GMPR | na |  |  | 11218 | -0.126 | -0.3661 | No |
| 39 | RIPK2 | na |  |  | 11399 | -0.146 | -0.3739 | No |
| 40 | SP110 | na |  |  | 11857 | -0.213 | -0.3954 | No |
| 41 | IL4R | na |  |  | 12312 | -0.263 | -0.4163 | No |
| 42 | PROCR | na |  |  | 12401 | -0.272 | -0.4178 | No |
| 43 | CASP1 | na |  |  | 12497 | -0.286 | -0.4196 | No |
| 44 | IL15 | na |  |  | 12671 | -0.309 | -0.4252 | No |
| 45 | LAMP3 | na |  |  | 13571 | -0.423 | -0.4675 | No |
| 46 | IRF9 | na |  |  | 13794 | -0.458 | -0.4739 | No |
| 47 | IFITM1 | na |  |  | 13940 | -0.477 | -0.4762 | No |
| 48 | PARP14 | na |  |  | 13951 | -0.479 | -0.4713 | No |
| 49 | IFIT3 | na |  |  | 13997 | -0.489 | -0.4682 | No |
| 50 | PLSCR1 | na |  |  | 14338 | -0.526 | -0.4801 | No |
| 51 | RTP4 | na |  |  | 14707 | -0.591 | -0.4927 | No |
| 52 | TDRD7 | na |  |  | 14836 | -0.610 | -0.4926 | No |
| 53 | CXCL10 | na |  |  | 14952 | -0.628 | -0.4915 | No |
| 54 | PSMA3 | na |  |  | 15431 | -0.706 | -0.5086 | No |
| 55 | NUB1 | na |  |  | 15548 | -0.732 | -0.5065 | No |
| 56 | EIF2AK2 | na |  |  | 16039 | -0.830 | -0.5228 | Yes |
| 57 | NCOA7 | na |  |  | 16217 | -0.875 | -0.5222 | Yes |
| 58 | LY6E | na |  |  | 16278 | -0.893 | -0.5153 | Yes |
| 59 | TRIM21 | na |  |  | 16298 | -0.897 | -0.5063 | Yes |
| 60 | LGALS3BP | na |  |  | 16379 | -0.918 | -0.5001 | Yes |
| 61 | TRIM5 | na |  |  | 16674 | -0.993 | -0.5044 | Yes |
| 62 | IFITM3 | na |  |  | 16890 | -1.051 | -0.5038 | Yes |
| 63 | TRAFD1 | na |  |  | 16979 | -1.082 | -0.4963 | Yes |
| 64 | TAP1 | na |  |  | 16995 | -1.089 | -0.4849 | Yes |
| 65 | LAP3 | na |  |  | 17397 | -1.213 | -0.4922 | Yes |
| 66 | WARS | na |  |  | 17398 | -1.213 | -0.4786 | Yes |
| 67 | PARP9 | na |  |  | 17466 | -1.243 | -0.4682 | Yes |
| 68 | ELF1 | na |  |  | 17482 | -1.252 | -0.4549 | Yes |
| 69 | IFITM2 | na |  |  | 17505 | -1.262 | -0.4419 | Yes |
| 70 | UBE2L6 | na |  |  | 17509 | -1.262 | -0.4279 | Yes |
| 71 | PSME2 | na |  |  | 17817 | -1.395 | -0.4283 | Yes |
| 72 | ISG20 | na |  |  | 17840 | -1.410 | -0.4136 | Yes |
| 73 | FAM46A | na |  |  | 17904 | -1.443 | -0.4007 | Yes |
| 74 | BATF2 | na |  |  | 17919 | -1.450 | -0.3852 | Yes |
| 75 | HLA-C | na |  |  | 17936 | -1.459 | -0.3696 | Yes |
| 76 | CD74 | na |  |  | 17961 | -1.469 | -0.3544 | Yes |
| 77 | IRF2 | na |  |  | 17974 | -1.475 | -0.3385 | Yes |
| 78 | PSMB8 | na |  |  | 18223 | -1.614 | -0.3333 | Yes |
| 79 | NMI | na |  |  | 18304 | -1.687 | -0.3186 | Yes |
| 80 | CCRL2 | na |  |  | 18863 | -2.300 | -0.3220 | Yes |
| 81 | PSMB9 | na |  |  | 18892 | -2.341 | -0.2972 | Yes |
| 82 | IFI27 | na |  |  | 18923 | -2.408 | -0.2717 | Yes |
| 83 | BST2 | na |  |  | 18934 | -2.418 | -0.2451 | Yes |
| 84 | TXNIP | na |  |  | 18972 | -2.510 | -0.2188 | Yes |
| 85 | GBP2 | na |  |  | 19029 | -2.666 | -0.1919 | Yes |
| 86 | GBP4 | na |  |  | 19050 | -2.740 | -0.1621 | Yes |
| 87 | CXCL11 | na |  |  | 19056 | -2.745 | -0.1316 | Yes |
| 88 | PSME1 | na |  |  | 19078 | -2.824 | -0.1010 | Yes |
| 89 | CD47 | na |  |  | 19123 | -3.029 | -0.0693 | Yes |
| 90 | B2M | na |  |  | 19139 | -3.164 | -0.0346 | Yes |
| 91 | C1S | na |  |  | 19156 | -3.351 | 0.0022 | Yes |
Table: GSEA details [plain text format]

  

Fig 2: HALLMARK\_INTERFERON\_ALPHA\_RESPONSE      
 Blue-Pink O' Gram in the Space of the Analyzed GeneSet

  

Fig 3: HALLMARK\_INTERFERON\_ALPHA\_RESPONSE: Random ES distribution      
 Gene set null distribution of ES for **HALLMARK\_INTERFERON\_ALPHA\_RESPONSE**
